# Chronic Pain in the Emergency Department: A Pilot Mixed-Methods Cross-Sectional Study Examining Patient Characteristics and Reasons for Presentations

**DOI:** 10.1155/2016/3092391

**Published:** 2016-10-18

**Authors:** Patricia A. Poulin, Jennifer Nelli, Steven Tremblay, Rebecca Small, Myka B. Caluyong, Jeffrey Freeman, Heather Romanow, Yehudis Stokes, Tia Carpino, Amanda Carson, Yaadwinder Shergill, Ian G. Stiell, Monica Taljaard, Howard Nathan, Catherine E. Smyth

**Affiliations:** ^1^The Ottawa Hospital Research Institute, Ottawa, ON, Canada; ^2^Department of Anesthesiology, University of Ottawa, Ottawa, ON, Canada; ^3^The Ottawa Hospital Department of Psychology, Ottawa, ON, Canada; ^4^Faculty of Medicine, Memorial University, St. John's, NL, Canada; ^5^Department of Emergency Medicine, Faculty of Medicine, University of Ottawa, Ottawa, ON, Canada; ^6^Children's Hospital of Eastern Ontario, Ottawa, ON, Canada; ^7^Carleton University, Ottawa, ON, Canada; ^8^York University, Toronto, ON, Canada; ^9^School of Epidemiology, Public Health and Preventative Medicine, University of Ottawa, Ottawa, ON, Canada

## Abstract

*Background*. Chronic pain (CP) accounts for 10–16% of emergency department (ED) visits, contributing to ED overcrowding and leading to adverse events.* Objectives*. To describe patients with CP attending the ED and identify factors contributing to their visit.* Methods*. We used a mixed-method design combining interviews and questionnaires addressing pain, psychological distress, signs of opioid misuse, and disability. Participants were adults who attended the EDs of a large academic tertiary care center for their CP problem.* Results*. Fifty-eight patients (66% women; mean age 46.5, SD = 16.9) completed the study. The most frequently cited reason (60%) for ED visits was inability to cope with pain. Mental health problems were common, including depression (61%) and anxiety (45%). Participants had questions about the etiology of their pain, concerns about severe pain-related impairment, and problems with medication renewals or efficacy and sometimes felt invalidated in the ED. Although most participants had a primary care physician, the ED was seen as the only or best option when pain became unmanageable.* Conclusions*. Patients with CP visiting the ED often present with complex difficulties that cannot be addressed in the ED. Better access to interdisciplinary pain treatment is needed to reduce the burden of CP on the ED.

## 1. Introduction

 Chronic pain (CP) is defined as pain that persists for more than 3 to 6 months or beyond the normal healing time [1] and it affects between 19 and 30% of the population in the USA, Canada, and Europe [2–4]. Chronic pain accounts for 10–16% of emergency department (ED) visits [5, 6] with some patients relying heavily on emergency resources [7, 8]. The ED is not considered an appropriate setting for treating chronic pain [5, 7, 9, 10] and it is well established that many ED visits for chronic diseases are preventable [11]. While life-threatening exacerbations of chronic illnesses do require emergency attention, in 2014-2015, there were more than 4.3 million ED visits that were classified as level 4 (less urgent) or level 5 (nonurgent). The use of the ED for nonurgent medical conditions such as chronic pain puts undue pressure on ED resources [9, 12, 13] and on the healthcare system given that ED visits cost about 5 times a general practitioner visit [14]. Furthermore, the use of the ED may result in inconsistent or inappropriate care as well as adverse events [5, 15, 16]. For all of these reasons, many jurisdictions are working to develop and evaluate programs designed to improve the quality of care and reduce the use of acute care resources for patients with chronic conditions [17] such as diabetes [18] or chronic obstructive pulmonary disease [19, 20].

To effect solutions for patients with chronic pain who use the ED, we first need to understand who they are, the factors contributing to their presentation, and their opinion on how to improve their pain and functioning. Previous studies have examined physician and ED staff attitudes towards patients with chronic pain [21], barriers to providing care [9], and opiate abuse indicators [22]. This study focuses on the medical and psychosocial characteristics of patients presenting to the ED with chronic pain along with their perceived needs. The objectives of this study are to (1) describe the medical and demographic characteristics of patients with chronic pain presenting to the ED and determine statistical parameters to plan future studies; (2) identify precipitating factors contributing to the use of the ED; and (3) determine patients' perceived needs regarding possible alternatives to using the ED. This information will assist us in developing an interventional study for ED patients with chronic pain and will also provide ED physicians and staff with a more comprehensive clinical picture of the chronic pain patients they encounter.

## 2. Methods

### 2.1. Study Design

We used a mixed-methods study design and administered a series of validated questionnaires as well as semistructured interviews to a convenience sample of patients with chronic pain who presented to the ED during the months of July and August 2013. All procedures contributing to this work comply with the ethical standards of the relevant national and institutional committees on human experimentation and with the Helsinki Declaration of 1975, as revised in 2008. The study was approved by the Ottawa Health Science Network Research Ethics Board (OHSN-REB).

### 2.2. Setting

Participants were recruited through the two EDs of the Ottawa Hospital (Ottawa, Ontario, Canada), a large Canadian academic tertiary care centre (1,127 beds) with approximately 166,000 annual visits [23].

### 2.3. Participant Inclusion Criteria

To be eligible for the study, participants had to be 18 years of age or older, English or French speaking, with a primary complaint of pain meeting our predefined criteria for chronicity. The pain could be constant (e.g., low back pain, fibromyalgia) or recurrent (e.g., migraine, nephrolithiasis) but had to have been present for longer than three consecutive months. Patients were excluded if they had communication barriers, had a medical condition listed as critical, had suffered an assault, had evidence of acute intoxication, or were unable to provide consent.

### 2.4. Procedures

ED staff and physicians identified patients meeting the inclusion criteria during thirty ED shifts. These shifts were randomly selected using computer-generated random numbers. Patients who met inclusion criteria were informed of the study by ED staff at the time of their visit. One of two senior anesthesiology residents (JN or ST) from the research team met with patients who expressed interest in participating in the study to give further details and obtain informed consent before conducting the interviews and collecting the survey data. This was done while the patient was waiting to be seen by the ED physician. Interviews generally lasted between 10 and 30 minutes.

### 2.5. Data Collection

The following demographic information was collected: age, sex, ethnicity, education level, employment status, number of people in home, family income, healthcare problems, current medications, and health insurance. Interviewers used a semistructured interview format and open-ended questions to elicit participants' perspectives on their primary pain problem and the circumstances surrounding their visit to the ED. After the interviews, patients completed the following instruments: Brief Pain Inventory (BPI) [24], the Pain Catastrophizing Scale (PCS) [25], the Screener and Opioid Assessment for Patients with Pain-Revised (SOAPP-R) [26], the Patient Health Questionnaire-9 (PHQ-9) [27], the Generalized Anxiety Disorder-7 (GAD-7) [28], the Post-Traumatic Stress Disorder Checklist-Civilian Version (PCL-C) [29], and the Insomnia Severity Index (ISI) [30]. They were also asked to complete a checklist about their perceived needs regarding possible alternatives to visiting the ED for their chronic pain.

All the data collection was performed by the same two anesthesiology residents who obtained informed consent from participants. They were not involved in the care of the patients. The residents both had prior research experience and participated in study-specific training that involved discussions of qualitative research and interviewing. The interview preceded the administration of the questionnaires to avoid undue influence on patients' responses to the interviews.

### 2.6. Data Analysis

Demographic characteristics of patients with chronic pain were described using means and standard deviations for continuous variables and frequencies and percentages for categorical variables. Indicators of health services utilization (number of ED visits, hospital admissions, and length of stay) were described using mean and standard deviation, as well as range, median, and interquartile range (Q1–Q3) to account for skewness. All statistical analyses were performed using the software Statistical Package for the Social Sciences version 20 [31]. At this early stage in attempting to understand use of the ED by patients with chronic pain, we did not have specific hypotheses to test. As such, the sample size was determined primarily based on logistical considerations. However, we determined that a sample size of 58 patients was adequate to meet our objectives for this pilot study: first, using a standard deviation estimate for the BPI Pain Interference scale of 2.5 from a previous study conducted at our centre, this sample size is adequate to estimate the mean BPI Pain Interference score using a two-sided 95% confidence interval with a total width of no more than 1.3. Second, this sample size would allow us to reliably estimate the standard deviation, an important parameter required for sample size calculation in a future trial. In particular, Browne [32] suggested that using the upper limit of the 95% confidence interval of the population variance in the formula for sample size calculation would guarantee the planned power with a probability of at least 0.95. Our sample size is adequate to estimate the population standard deviation with a total width of no greater than 1, that is, a lower limit of 2 and an upper limit of 3 [33]. Finally, this sample size is sufficient for the qualitative portion of our study [34].

The audio-recorded interview data were transcribed verbatim, checked, and entered into NVivo 10 [35], a qualitative analysis software program. An inductive thematic analysis was used to analyse the data [36, 37]. Each individual transcript was read in its entirety to gain an understanding of the interview as a whole. The analysis was directed towards the objectives of the inquiry. Segments of the data (entire response, sentence, or words) were coded accordingly. Subsequently, the codes were sorted into themes relevant to the research questions, defined and named prior to detailed analysis. Finally, examples were extracted from the interviews to illustrate each theme. The analysis of the data was completed independently by two raters (TC and YSt) and then compared and discussed with the first and senior authors to ensure consistency of the coding and interpretation.

## 3. Results

Sixty-five patients meeting inclusion criteria were referred to the study, of whom 58 (89%) agreed to participate and completed the entire study. This accounts for approximately 2.5% of all patients registered in the ED during the included shifts. Descriptive characteristics of participants can be found in Table 1.

Participants were predominantly Caucasian (79%), middle aged (M = 46.5, SD = 16.9), and women (66%), with at least some college or university education (71%). They had, on average, been living with chronic pain for 5 years (Q1–Q3 = 2.4 to 13.0 years) and reported moderate or severe levels of pain intensity (M = 6.8, SD = 1.7) and pain interference (M = 7.4, SD = 2.2).

The most commonly cited reasons for visiting the ED were inability to cope with pain (60%), worry about what was causing the pain (16%), or receiving advice from a physician (10%). Forty-nine (85%) of the 58 participants had a primary care provider who they had visited, on average, 10.2 times (SD = 10.2, median = 6.5) over the previous 12 months. Forty-six participants (79%) had visited the ED for the same problem in the previous 12 months (Mean number of visits = 5.4, SD = 10.0, median = 1), with one participant having visited the ED 55 times. Sixteen (27%) participants had been hospitalized for their pain in the past 12 months, with one participant having 20 admissions for chronic pain (Mean number of admissions = 2.9, SD = 4.7, median = 1; median length of stay = 8 days, maximum = 105 days). Forty-two (72%) participants were taking opioids (see Table 2).

Psychosocial trauma and mental health problems were common. Fifty-three percent (*n* = 31) of participants reported a history of physical, sexual, or emotional abuse. Thirty-six (62%) participants scored within the range of moderate to severe depression, 25 (43%) participants reported moderate or severe anxiety, 28 (48%) participants reported symptoms suggesting posttraumatic stress disorder, and 23 (40%) scored above the cut-off predicting risk of aberrant drug-related behaviours as assessed by the SOAPP-R questionnaire (see Table 3). Only 3 (5%) of the participants had seen psychiatrists at some point for management of their concomitant mental health problems. In terms of coping with pain, more than half of all participants had high levels of pain catastrophizing (M = 30.1, SD = 14.5).

In addition to their chronic pain, participants had several medical problems: 53% had more than three medical conditions, the most frequent being asthma (33%), arthritis (28%), hypertension (28%), and diabetes (20.7%). Seventy-two percent (72%) had seen other physicians including cardiology, gastroenterology, neurology, neurosurgery, gynecology, and urology looking for a remediable cause and treatment for their chronic pain. Six (10%) participants had seen a pain specialist at some point for their chronic pain.

When asked to choose from a list of potential options for better chronic pain management, 30 (52%) participants were interested in more effective medications, 27 (47%) participants were interested in having a healthcare professional explain their pain, and 23 (40%) were interested in a referral to a pain clinic. There was also some interest in an exercise/rehabilitation program (21%), surgery (19%), counselling/peer support/nondrug approach (14%), and decreasing or stopping medications (5%).

A thematic analysis of the interview transcripts revealed several important themes concerned with both the participants' chronic pain and their experience in the ED. The following are quotes from participants within each theme.

*Thematic Analyses: Quotes from Participants' Interviews*

*Uncontrollable Pain*

*It gets to the point where it feels like I'm going to die sometimes.*

*It was unbearable.*

*…once it gets too bad, and I have to double on the pills that's it, I have to come in.*

*Last night it was continuous pain. I couldn't sleep, so I said to my husband, ‘I think I better go to the emerg.'*

*Inability to Function*

*I couldn't keep nothing down today, and my husband was literally freaking out because I had no liquids today… My migraine headaches are really bad, and when they're bad, I can't keep anything down … So that's why I'm here.*

*I got a headache and I couldn't see. I get up to walk and I was stumbling around.*

*I was going to get back on the sofa, and I couldn't move, couldn't move at all, my legs, my arms…The ambulance had to come and they got me on a stretcher.*

*Limited Primary Care Provider Effectiveness and Accessibility*

*He's just basically giving me meds and not trying to fix the problem.*

*I don't think she's doing everything that she can.*

*I find my doctor unsympathetic … When I went to her, she didn't want to listen to me talking about the pressure around my ear. She said to me, ‘For this session, that's all I have the time for,' and I haven't been called back … And there was no talk of pain management or anything. So I thought to myself that I can't rely on her.*

*Seeking Answers*

*I just want to know what's going on and what I can do, like what they could do … I just want to know what I have and deal with that. *

*Everything is numb. What am I waiting for? What do I have, cancer or what? ‘Cause my doctor told me I might have cancer. If I have cancer, at least I gotta know what I have. *

*Seeking Pharmacological Treatment*

*Um, basically I have chronic pain all of the time, and there was a mix up with one of my prescriptions and basically, I'm without any pain medication now. *

*Usually I have certain medications that I take. But then after a certain amount, I'm no longer allowed to take it, so I have to come to the emergency because first, the medication is not working, and second, I feel so nauseated that I can't continue to do my daily routine, so I really need to get something fast, like an IV.*

*Feeling Invalidated or Labelled as Drug Seeking in the Emergency Department*

*And I said, like, ‘Ow, ow, ow,' you know. And he said, ‘Well, you're not crying or screaming bloody murder, so I don't think you have fibromyalgia.*

*I said I'm not here for a prescription. I'm not. I'm just here to get the back pain under control. And he [the physician] just walked out. *

*I kept showing them, “This is the bottle of morphine prescribed back in May. Look! It's almost all here. I'm not taking it.” Like I felt I had to defend … I was very insulted.*

The most frequently cited motivation for attending the ED was uncontrollable pain. Patients described their pain as “*unbearable*,” “*overwhelming*,” and “*uncontrollable*,” often leading to feelings of desperation. As a result, many participants stated that they were “*not able to cope*” with their chronic pain and believed there were no other options available but to present to the ED. In addition to uncontrollable pain, patients reported a high degree of pain interference leading to an inability to sleep, eat, or walk that prompted them to come to the emergency department. When asked about their primary care providers, the majority stated that they had a provider that they felt comfortable discussing their pain problems with, but they noted limited time availability (e.g., holidays, not enough time to address concerns in one session) or limited emotional availability. Many also reported that their primary care physician was not effective in managing their pain. Some patients also reported that they had decided to visit the emergency room to access pharmacological treatment (e.g., prescription renewal or medication through intravenous route) or to seek answers about the cause of their pain problem, fearing that something serious might be underlying their pain. Finally, although this experience was not universal, several patients commented on their actual experience accessing the emergency room for their pain problem and reported feeling invalidated or labelled as a drug seeker.

## 4. Discussion

The objective of this study was to understand factors contributing to the use of the ED for chronic pain, to describe the medical and psychosocial characteristics of patients with chronic pain who use the ED, and to inquire about patients' perspective on potential alternatives to ED visits.

The most common reason why study participants visited the ED was inability to cope with their pain. This was reflected in both the patient interviews and the survey responses. Insufficient coping has been associated with increased pain, depression, disability, and poor psychological adjustment [38]. A lack of coping strategies may also explain why chronic pain patients seek help from the ED. For example, pain catastrophizing has been identified as a maladaptive coping strategy. It is defined by ruminations, helplessness, and magnification of pain symptoms; some patients have high fear of their pain symptoms and may entertain the beliefs that the current situation will lead to the worst possible outcome [39]. We found that many patients presenting to the ED with chronic pain had high rates of pain catastrophizing. Maladaptive coping strategies such as pain catastrophizing not only lead to greater disability, pain intensity [40], decreased quality of life, and lower exercise capacity, but also may encourage the increased utilization of acute care services. Fortunately, there are effective interventions such as cognitive-behavioural therapy [41], acceptance and commitment therapy, or mindfulness-based stress reduction [39] that can assist patients in feeling less helpless and fearful towards their pain and refocus their attention on other areas of their lives. However, these treatments are beyond the scope of an ED intervention and often difficult to access.

Participants suffered from mental health problems at a rate significantly higher than that reported in the general population, as found in other studies [15, 42]. More than half of the participants endorsed moderate or severe depressive symptoms compared to 3.8% of the general population [43]. More than 40% endorsed moderate or severe anxiety symptoms compared to approximately 4% in the general population [44]. Symptoms of PTSD were also common and over 50% of the participants endorsed having experienced verbal, physical, or sexual trauma. The high rate of depression and anxiety is consistent with observations in pain clinic patients [45].

Substance use-related issues are another potential factor driving ED visits; while the instrument we used is not a diagnostic tool for an opioid-use disorder, 40% of the participants scored in the clinically relevant range suggesting a risk of opioid aberrancy. This is important given that 72% of the patients seen were on opioids and that some patients shared with the interviewer that they came to the ED for prescription renewals even though this is against the policy of the ED where the study was conducted.

It is interesting that mental health problems did not emerge as a theme in the interviews and may reflect patients' fear that their pain could be deemed “psychological.” Alternatively, it may represent a lack of awareness of the role that anxiety, depression, trauma, and addiction play in chronic pain. Unrecognized and untreated mental illness can significantly impede successful rehabilitation of chronic pain patients [46]. Mental illness may also increase pain intensity and perpetuate pain-related physical dysfunction [47]. To successfully manage chronic pain, there must be concurrent diagnosis and treatment of mental illness. However, given the acute care focus of the ED, specific attention to mental health issues in the management of chronic pain is rarely possible.

More than half of our participants had at least three medical comorbidities. It is challenging for healthcare professionals in the ED with limited time and incomplete medical and medication histories to identify and prioritize the relative contributions of medical issues, mental health, and substance dependence to the chronic pain complaint. There are safety concerns as well because prescribing opioids for chronic pain in the ED without a thorough knowledge of the patient may increase the risk of adverse drug-related events and complications, including death [16, 48].

In contrast to previous studies showing an association between ED use and lack of access to a primary care physician [9], we found that the majority of patients visiting the ED had a primary care physician that they visited regularly. The interviews with participants offered some insight into the reasons why they turned to the ED rather than to their primary care provider; many patients felt their primary care provider was ineffective in treating their chronic pain or was not accessible in a timely manner. Many also reported that while they felt their primary care provider was doing their best to address their pain problem, it was still insufficient for their needs. Several factors might contribute to this perception; in general, many primary care providers feel uncomfortable managing chronic pain, rating chronic pain second only to mental illness as a clinically challenging area [49, 50]. Physicians receive little or no formal training on pain management [51], are fearful of opioid abuse or diversion [52], worry about the scrutiny of their practice by regulatory bodies [53], and have significant problems accessing expert opinion and support [54]. While most chronic pain can and should be managed in the community, there is an unmet need for individuals with more complex disease. Unfortunately, publicly funded interdisciplinary programs, which are the gold standard in the treatment of chronic pain [55], continue to be difficult to access [56]. It is thus not surprising that patients with access to primary care still continue to use the ED for management of their chronic pain.

Finally, we found that chronic pain patients who present to the ED are generally interested in other methods to better manage their symptoms. Although the majority of participants (52%) stated they were most interested in more effective medications to manage their pain, 40% of the participants were interested in being referred to a pain clinic and 47% were interested in having a health professional explain their pain condition to them. It would be beneficial to explore and test these options in a focused program for patients who frequently visit the ED for their chronic pain; some options include rapid access to education about pain, self-management training, and interventions designed to address depression, insomnia, and anxiety which may have a beneficial impact on patient pain experience and function.

## 5. Limitations

Our study has several limitations. The most important ones are that we did not set out to select a probability sample, that our sample is small, and that we only recruited patients from one hospital. We were able to interview, on average, 2 patients with chronic pain per ED shift. Given that, at our institution, 10.4% of patients presenting to the ED do so for a chronic pain problem [57] and that the ED sees on average 152 patients per shift, this represents only 12.5% of the population of chronic pain patients who visit the ED. For these reasons, our participants may not be representative of the population of patients with chronic pain who seek services in the ED. Conducting research in the ED presents several challenges and it was not possible for us to screen consecutive patients for eligibility. We relied on ED staff to identify potential participants and staff participation may have varied with ED occupancy. Screening of charts to ascertain how many patients might have met inclusion criteria was complicated by inconsistent documentation of a chronic pain disorder. Recruitment bias may have occurred as certain patients may have been identified by ED staff based on features of their presentation or history that made them stand out.

Another limitation is that we elicited patients' perspective on potential alternatives to ED visits through a checklist of items that were derived from clinical interactions with patients. Other strategies to elicit information could involve a focus group that would provide opportunities to discuss pros and cons and applicability and potentially come up with other creative ideas to implement and evaluate.

Finally, we had a higher than average number of female, well-educated Caucasians. Chronic pain is more prevalent among women and women are less likely to have pain optimally treated [58]. There are notable differences in the chronic pain experience among different genders, cultures, and ethnicities [59]. However, the themes of medical and psychiatric comorbidities, maladaptive coping, and lack of effective care in the community are likely constant across most populations as reflected in the general pain literature.

## 6. Future Direction

Our study has demonstrated that even though the majority of participants were, in fact, under the care of a primary care provider, they still chose to come to the ED for their pain problem. It appears that their complex pain, medical, and psychosocial needs are not being met within the community. Further research is needed to understand the nature of the relationship between the patient and their primary care provider and to identify and address possible barriers that prevent patients from seeking help for their pain problem from them. We also need to examine how primary care physicians could become more effective in addressing chronic pain in their practice as well as what resources are required to meet the needs of patients in the community. This could include improving access to evidence-based interventions for chronic pain including cognitive-behavioural therapy, mindfulness-based programs, support groups, self-management programs, or interdisciplinary management to improve clinical outcomes and reduce acute care use among patients with chronic pain.

Further work is also required to identify the biopsychosocial characteristics of patients in other centres to examine regional differences if there are any. Focusing also on patients with chronic pain who are heavy users of the ED may allow opportunities to design effective interventions that have the potential to not only improve clinical outcomes but also reduce healthcare costs. There is also an urgent need to evaluate different strategies for addressing chronic pain in the ED. This could be accomplished by providing more training for ED physicians on chronic pain and substance use disorders and their treatments as well as addressing factors contributing to nonadherence to guidelines surrounding the use of opioids in chronic noncancer pain. Having ED physician better prepared to care for patients with chronic pain may improve the experience of physicians, staff, and patients and lead to better clinical outcomes and rational use of urgent care resources. However, solving the issue of chronic pain in the ED requires the involvement of all interested stakeholders and we believe that an important step in this work is to bring together patients, clinicians, caregivers, and decision-makers to come up with solutions that will lead to an improved health system.

## 7. Conclusion

Patients with chronic pain require more than what the emergency department can offer. What is unique to this investigation, benefitted by the mixed-methods methodology, is a clearer picture of the reasons why patients with chronic pain require a more multifaceted and longitudinal approach to care than an ED can deliver.

The complex nature of chronic pain, in association with the high incidence of mental health issues and comorbid medical conditions seen in this group, makes the fast-paced assessment and treatment models used in the ED suboptimal for their management. Understanding the factors that bring chronic pain patients to the ED may lead to a better use of healthcare resources and improve treatment outcomes. Based on our study, we believe that interventions should target adaptive coping strategies and address the high levels of depression, anxiety, insomnia, and PTSD seen in this group. Patients who present to the ED with chronic pain have multiple mental and physical challenges that, in some cases, have not been adequately addressed by their primary care providers. We are currently testing whether access to a multidisciplinary team linked to primary care can improve clinical outcomes and reduce ED visits in this population.

## Figures and Tables

**Table 1:** Characteristics of 58 patients who visited the emergency department for chronic pain.

Characteristics	Mean	SD
Age (years)	46.5	16.9

	*N*	%

Gender		
Male	20	34.5
Female	38	65.5
Marital status		
Single (never married)	17	29.3
Single (separated or divorced)	8	13.8
Married or living with partner	31	53.4
Widowed	2	3.4
Ethnicity		
Caucasian	46	79.3
First Nations	1	1.7
Asian	2	3.4
African	3	5.2
Other	6	10.3
Education level		
Less than grade 12	10	17.2
High school diploma	7	12.1
Some college or university	16	27.6
College or university degree	17	29.3
Postgrad/professional degree	8	13.8
Number of dependents		
0	2	3.4
1	13	22.4
2	17	29.3
3	9	15.5
4 or more	12	20.7
Family income		
Less than $20,000	14	24.1
$20,000–$29,999	3	5.2
$30,000–$49,999	5	8.6
$50,000–$69,999	7	12.1
$70,000 or more	14	24.1
Did not say	15	25.9
Employment status before pain		
Full time	30	51.7
Part time	3	5.2
Retired	10	17.2
Sick leave/disability	4	6.9
Other	11	19.0
Current employment status		
Full time	12	20.7
Part time	2	3.4
Retired	11	19.0
Sick leave/disability	24	41.4
Other	9	15.4
Insurance coverage		
None	13	22.4
Government	14	24.1
Third party	31	53.4

**(a):** 

	*N*	%
Location		
Lower back	18	31.0
Abdomen	11	19.0
Joint pain	8	13.8
Headache/migraine	7	12.1
Leg	5	8.6
Pelvic/genital	4	6.9
Chest	3	5.2
Neck	2	3.4
Use of any opioid^*∗*^	42	72.4
Strong opioids (oxycodone, morphine, hydromorphone, etc.).	32	55.2
Weak opioids (tramadol and codeine)	24	41.4

^*∗*^Please note that some participants were on more than one medication.

**(b):** 

Access to PCP (% with a family physician)	84.5%
Number of PCP visits for pain in 12 months prior to study visit (mean, SD)	8.1 (10.2)
Median (Q1–Q3)	4 (1–12)
Range	0–50
Number of visits to the ED 12 months prior to study visit (mean, SD)	5.4 (10.0)
Median (Q1–Q3)	1 (0–4)
Range	0–55
Number of admissions for pain in the past 12 months prior to study visit (mean, SD)	2.9 (4.7)
Median (Q1–Q3)	1 (1–3)
Range	1–20
Hospitalization days for pain in the past 12 months prior to study (mean, SD)	14.9 (25.8)
Median (Q1–Q3)	8 (5–13)
Range	2–105

*Note.* PCP: primary care physician; ED: emergency department.

**Table 3:** Descriptive statistics for all outcome measures.

Outcome measure	All 58 patients	Patients with moderate or severe symptoms or above clinical cut-off
	Mean (SD; 95% CI)	Frequency (%)
Brief Pain Inventory (BPI)		
Average pain intensity	6.8 (1.7; 6.3–7.2)	46 (79.3%)^1^
Average pain interference	7.4 (2.2; 6.9–8.0)	48 (86.2%)^2^
Patient Health Questionnaire-9 (PHQ-9)	12.2 (7.1; 10.4–14.0)	33 (56.9%)^3^
Generalized Anxiety Disorder-7 (GAD-7)	9.1 (6.7; 7.4–10.8)	25 (43.1%)^4^
Insomnia Severity Index-7 (ISI-7)	13.3 (8.4; 11.1–15.5)	25 (43.1%)^5^
PTSD Checklist-Civilian Version (PCL-C)	33.9 (18.2; 28.4–38.1)	9 (15.5%)^6^
Pain Catastrophizing Scale (PCS)	30.1 (14.5; 25.9–33.8)	30 (51.7%)^7^
Screener and Opioid Assessment for Patients with Pain-Revised (SOAPP-R)	16.9 (11.2; 13.7–18.45)	23 (39.7%)^8^

^1^BPI pain intensity ratings: moderate pain cut-off = 5 (47).

^2^BPI Pain Interference: moderate pain interference cut-off = 5 (47).

^3^PHQ-9 moderate depressive symptoms cut-off = 10 (21).

^4^GAD-7 moderate anxiety cut-off = 10 (22).

^5^ISI-7 moderate to severe insomnia cut-off = 15 (24).

^6^PCL-C cut-off suggestive of posttraumatic stress disorder = 50 (23).

^7^PCS cut-off indicating high levels of pain catastrophizing = 30 (19).

^8^SOAPP-R cut-off indicating increased risk of aberrant medication-related behaviours = 18 (20).

## Additional Points

*Summary*. Patients' inability to cope with pain is the primary reason for their presentation to the emergency department (ED). Mental health issues such as depression, anxiety, and symptoms of posttraumatic stress were common in this group. Although the majority of patients had a family physician, they did not see any other options to address their problem. The ED is not the ideal service for managing chronic pain patients' complex presentation. Better access to interdisciplinary programs is clearly needed to provide better care for patients with chronic pain and to reduce the burden on the ED.
